# Study on Ultrasonic Nondestructive Testing of Self-Compacting Concrete under Uniaxial Compression Test

**DOI:** 10.3390/ma15134412

**Published:** 2022-06-22

**Authors:** Yongshuai Sun, Guihe Wang, Yixuan Li

**Affiliations:** 1 College of Water Resources & Civil Engineering, China Agricultural University, Beijing 100083, China; 2 School of Engineering and Technology, China University of Geosciences, Beijing 100083, China; wanggh@cugb.edu.cn (G.W.); taiyuansys@163.com (Y.L.)

**Keywords:** self-compacting concrete, ultrasonic nondestructive testing, defects, uniaxial compression, pair measuring method

## Abstract

To study the variation law of ultrasonic parameters of self-compacting concrete before and after damage under uniaxial compression test conditions, the C30 self-compacting concrete blocks stored for 7 days and 28 days were subjected to ultrasonic nondestructive testing, and the variation law of the sound time, amplitude, and sound velocity before and after the damage of self-compacting concrete blocks was emphatically analyzed. The concrete acoustic detection software was introduced to judge and analyze the abnormal values of the parameters of each measuring point, and the defect distribution map of each test block was obtained. The results showed that after curing the self-compacting concrete test block for 7 days and 28 days, the average value of sound time before and after the failure of each measuring point of the test block is small, and the average value of sound time before the failure is less than that after; the average amplitude after failure is smaller than that before failure, and the amplitude of some measuring points will be smaller than that before. The average sound velocity after failure is less than that before failure, and the internal defects appear and the structure is not dense. This study provides a theoretical basis for the application of ultrasonic detection technology in the field of self-compacting concrete and also provides a practical basis for the stability monitoring and failure warning of self-compacting concrete.

## 1. Introduction

Self-compacting concrete (SCC) is a new composite material developed based on ordinary concrete; with its gravity, it can be compacted and formed, which has excellent construction performance [1,2,3,4,5]. In recent years, it has been widely used in many projects [6,7,8,9], which has become a new direction for the development of concrete materials. For example, a super-high-rise project adopted C60 SCC for pouring [10], and Miyun Reservoir adopted C20 SCC for the second lining project of a tunnel pipe [11], and it is applied in high-speed railway project construction [12] and in pouring prefabricated components [13].

Although SCC has good working performance, during the use of SCC, its structure is affected by an external load, and it undergoes internal defects and cavity initiation and expansion; finally, it leads to structural damage, which seriously affects the bearing capacity and durability of the components [14,15,16,17,18,19]. Therefore, we must choose appropriate methods to judge whether there are defects in the use of SCC materials to ensure the safety of concrete structures. The change of ultrasonic parameters before and after SCC failure is studied, to ensure that the quality defects of SCC components are detected without damage [20,21,22], which is the trend of SCC engineering quality detection.

Sandrine Rakotonarivo [23] studied the influence of concrete interface transition zone on ultrasonic parameters. In general, the construction period of concrete construction is long and the coverage is wide, which is easily affected by external factors [24,25]. Therefore, various defects are prone to occur in the actual pouring process, thus affecting the structural stability of concrete engineering. As a dynamic nondestructive testing method [26,27,28,29,30], ultrasonic testing technology has been widely used in the field of concrete due to its strong applicability, high detection sensitivity, and timely detection results [8]. In the detection of SCC engineering with defects [31,32,33], it can locate the defect position more accurately and further determine the causes of defects. The principle [31] of using ultrasonic detection technology to detect SCC in projects is that the ultrasonic pulse source emits a high-frequency elastic pulse wave to SCC, and then the wave fluctuation characteristics are recorded. When there is a discontinuous interface in concrete, the wave impedance surface appears on the defect surface, and when the wave reaches the interface, the transmission and reflection of the wave are produced, and the energy received by the wave is reduced [34]. When the concrete has serious defects such as looseness, honeycomb, and holes, it will produce scattering and diffraction of waves [35]. According to the initial arrival time of the wave and the energy attenuation characteristics of the wave, the frequency change, and the degree of waveform distortion, the density parameters of concrete can be obtained [36]. By processing and analyzing the ultrasonic characteristics of different sides and heights, the nature, size, and spatial relationship of concrete defects can be identified. It can be seen that, compared with the traditional detection technology, ultrasonic detection technology greatly improves the efficiency and accuracy of the whole project detection [37].

Scholars have studied the SCC engineering by ultrasonic testing. Dong Junfeng [8] used ultrasonic to test the void defects of self-compacting concrete and proposed a more accurate method to judge the sound velocity of void defects. Hao Wenxiu [38] compared the influence of different concrete strengths on ultrasonic wave speed. Gu Xingyu [39] established a three-phase finite element model of asphalt concrete concerning the results of ultrasonic testing. Huang Zhengyu [40] studied a simple and practical method for qualitative analysis of ultrasonic detection of concrete defects and imaging. Qu Xiushu [41] proposed the superposition principle in the calculation of the rectangular concrete-filled steel tube column based on the test data of the ultrasonic inspection of the concrete-filled steel tube. Zhao Guoqi [42] used ultrasonic signals in a specific frequency domain to perform health inspections on key parts of large concrete structures. Zheng Dan [43] studied the influence of frequency and water content on ultrasonic testing of concrete. Zhu Ziqiang [44] studied the attenuation characteristics of ultrasonic waves in concrete. Chen Dongdong [45] studied the power spectrum characteristics of ultrasonic wave propagation in concrete. Lin Weizheng [46] studied the thickness of cement concrete with the ultrasonic detector. Qin Tienan [47] measured the thickness of a concrete coating by the ultrasonic wave and evaluated the uncertainty. Petr Cikrle [48] introduced the application of ultrasonic inspection in concrete bridges and measured the size of holes in concrete panels.

In this paper, an ultrasonic testing analyzer was used to collect ultrasonic parameters of SCC before and after the uniaxial compression test, to study the variation of ultrasonic parameters of SCC before and after failure, to determine the distribution of internal defects in SCC under load. The evolution law of internal defects of SCC after failure is obtained, it provides a theoretical basis for the application of ultrasonic testing technology to the field of SCC, and it also provides a practical basis for stability monitoring and failure warning of SCC engineering.

## 2. Experiment Preparation

### 2.1. Test Raw Material and Proportion

The mixed design of SCC aims at the performance indexes needed in practical engineering [49]. The design code of SCC [50,51] and the experiment show that the working performance of the prepared concrete mixture meets the specified working requirements, and the designed proportioning scheme is scientific and feasible. Subsequent tests can be carried out with the prepared concrete [52,53]. Materials used in SCC mixtures are powders, natural aggregates, admixtures, and additives.

The cement selected is ordinary Portland cement with a grade of PO 42.5, the initial setting time is greater than 150 min, and the final setting time is less than 240 min. Natural aggregate mainly includes coarse aggregate and fine aggregate. The fine aggregate is river sand, the fineness modulus is 2.3–3.0, the main component is quartz sand, the surface is mostly round, the appearance is smooth, the texture is hard and dense, the porosity is low, the bonding force with cement is poor, the moisture content is 0.01%, and the water absorption is poor. The coarse aggregate is natural gravel, the particle size is 4.75–19 mm, the surface is rough and has the characteristics of porosity to absorb the cement slurry, and the bonding force with the cement is strong. The ratio of fine aggregate to coarse aggregate is 1.58. Admixture is a polycarboxylate water-reducing agent, and the main component is a polycarboxylate polymer masterbatch, with a high water-reducing rate, which can improve the fluidity of SCC by 25–35%, with good plasticity and being green and pollution-free. The performance indexes of raw materials used in the test are shown in Table 1.

**Figure 1:**
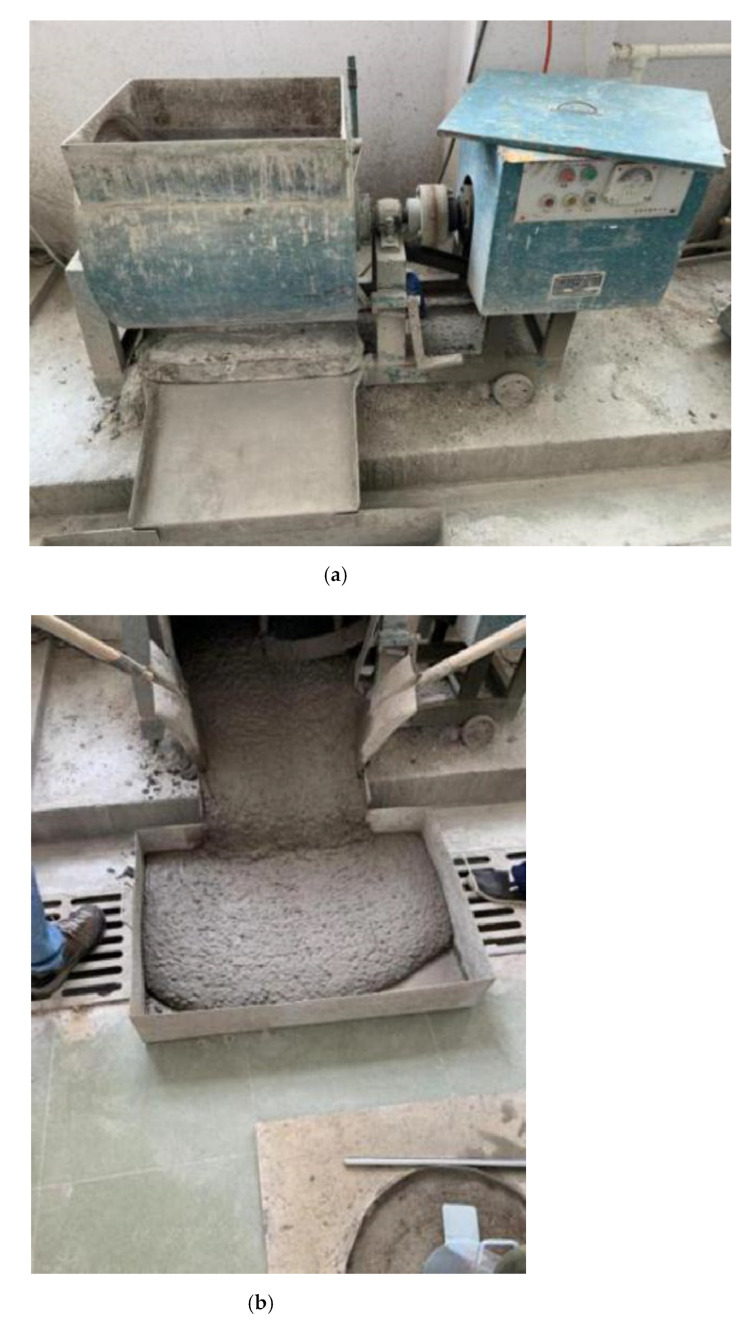
Photos of (**a**) single horizontal-axis forced concrete mixer and (**b**) finished SCC mixture.

According to the test requirements, C30 SCC was used for testing, and the ratios in Table 2 were obtained through multiple ratio tests. The SCC mix ratio is produced according to the design ratio. The quality of the required raw materials is weighed according to the design of each set ratio, and then crushed stone, fly ash, cement, and sand are added to the single-shaft forced concrete mixer in sequence (Figure 1a). Then, water and water reducer are added evenly during the mixing process, and the mixing process lasts for 3–5 min. After the mixing process is over, the mixer is turned off, the concrete mixture is placed upside down in the container, and the concrete is then mixed. The object is shown in Figure 1b.

**Figure 2:**
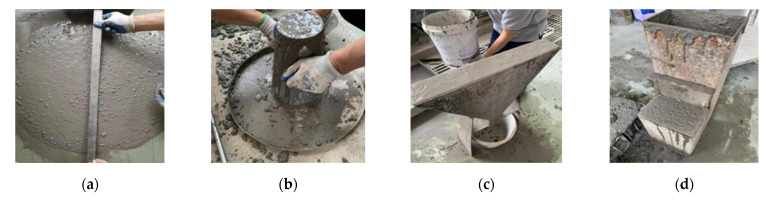
(**a**) Slump extension test; (**b**) extension time T500 test; (**c**) V funnel test; (**d**) L flow meter test.

We tested the slump extension, expansion time T500, v-shaped funnel time, and H2/H1 value of the concrete mixture [54]. Each test is shown in Figure 2.

See Table 3 for the work performance values and test results required by the SCC design code “Technical Specification for Self-compacting Concrete Application” JGJ/T 283-2012 and other requirements [55,56].

### 2.2. Specimen Design

Based on the design code of self-compacting concrete and the values obtained by tests [51,55,56,57], two groups of SCC test blocks (group A1 and group A2) with the specification of 150 mm × 150 mm × 150 mm were made, each as a set of three test blocks. After 24 h, the mold was removed and placed in the standard curing room for curing. Under the same curing conditions, the A1 test block was maintained for 7 days and the A2 test block was maintained for 28 days.

The experiment shows that the workability of the prepared concrete mixture meets the specified working requirements, and the designed mix proportion scheme is scientific and feasible. After that, tests can be carried out with the prepared SCC.

### 2.3. The Test Process

**Figure 3:**
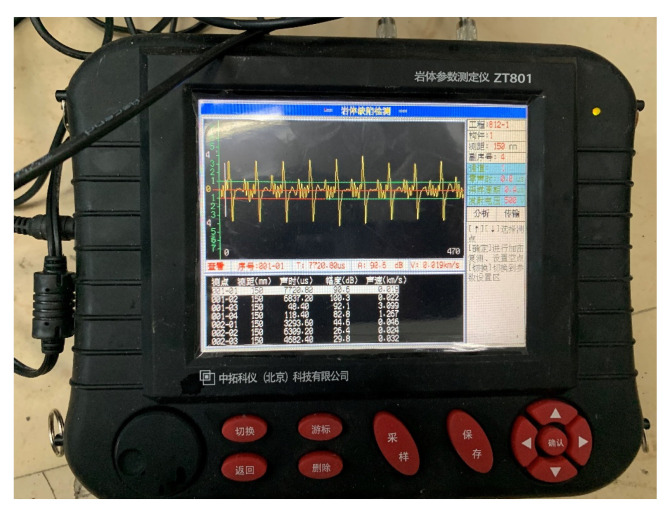
ZT801 Geotechnical Acoustic Tester.

Similar to acoustic emission detection [57], ultrasonic testing technology was used to detect each test block after curing. An ultrasonic testing analyzer (The ZT801 geotechnical acoustic wave tester produced by Zhongtuo Technology (Beijing) Technology Co., Ltd. was selected for this test.) was used to collect ultrasonic parameters of test blocks A1 and A2 before and after the uniaxial compression test. Before the uniaxial compressive strength test of the self-compacting concrete block, we used the ultrasonic testing analyzer (as shown in Figure 3) to sample the data from the intact test block, which is relatively close to the transmitting transducer and the receiving transducer. On the test point, for the accuracy of the test, we reduced the friction between the transducer and the test surface of the test block, reduced the loss of energy, and used the coupling agent to tightly fit the transducer on the test point.

Then, we collected the relevant measuring point data. The sampling sequence is carried out according to the arrangement order of the measuring point. The sampling method of each measuring point is the same. If there is an error in the sampling of a measuring point, the measurement point is remeasured, the acoustic parameters of each measurement point are collected multiple times, and the average value of the collected data is obtained. When the uniaxial compressive strength test of the self-compacting concrete is completed, the measurement point of each specimen after failure is sampled, and the sampling method is the same as the operation before failure. The data sampled by the geotechnical acoustic wave detection analyzer (The ZT801 geotechnical acoustic wave tester produced by Zhongtuo Technology (Beijing) Technology Co., Ltd. was selected for this test.) include the sound speed, sound time, and amplitude of each measuring point.

**Figure 4:**
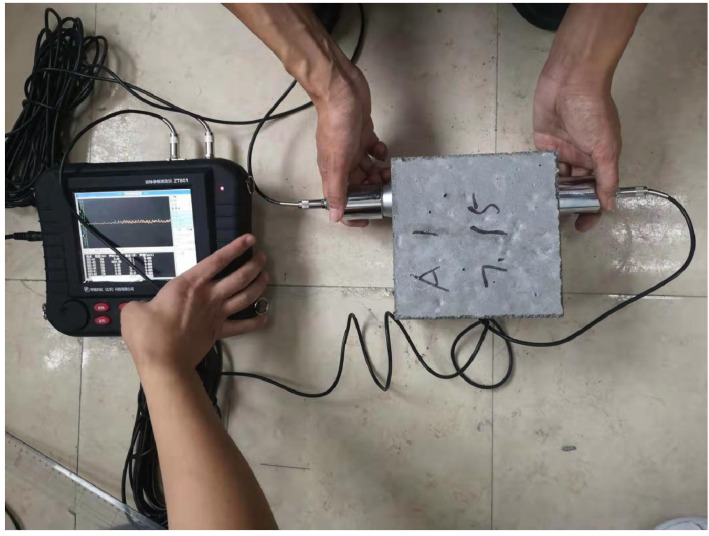
Pair measuring method.

**Figure 5:**
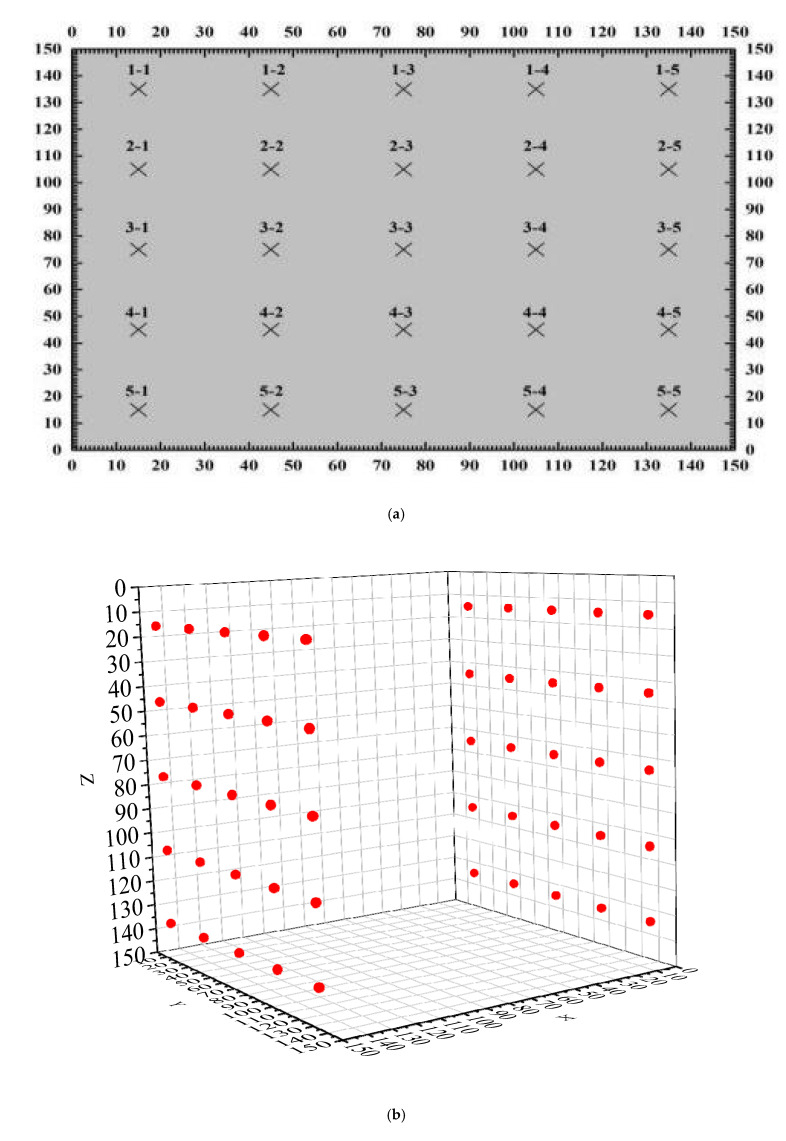
The layout of measuring points. (**a**) two-dimensional; (**b**) three-dimensional.

**Figure 6:**
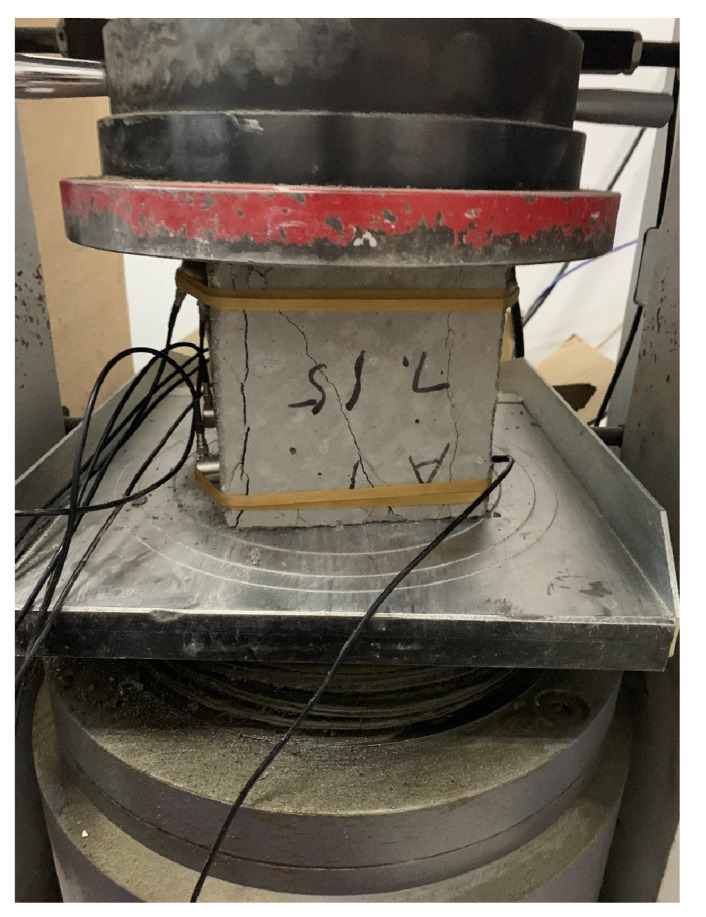
Pressure testing machine.

**Figure 7:**
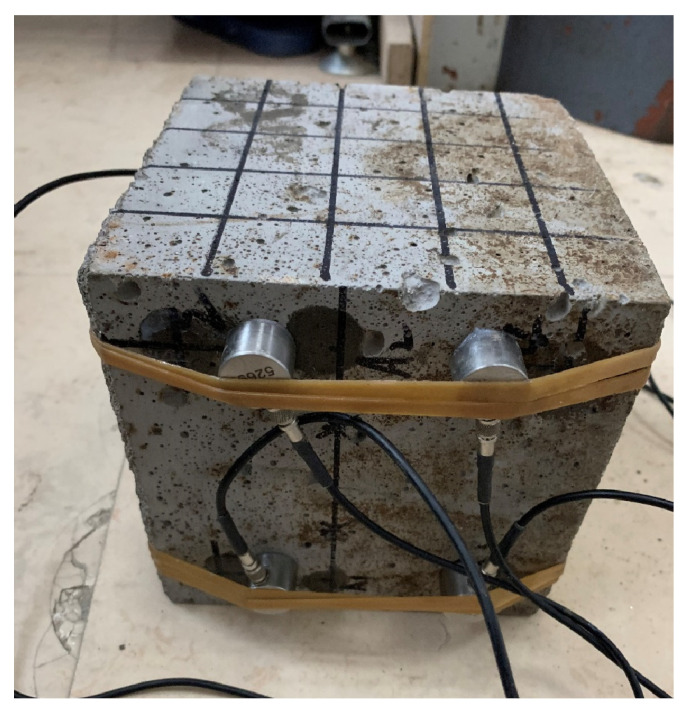
Sensor layout position.

For the experiment of pair measuring method (as shown in Figure 4) for data collection, the distance is 150 mm, the distance between measuring points is 0.25 m, the sampling period is 0.4 us, and the block surface is mesh of 5 × 5. We formed 25 test points, the detection of the surface relative to the surface, in the same position for 5 × 5 meshing, then formed, relatively, 25 test points, and the arrangement of the measuring points are shown in Figure 5. A uniaxial compression test was carried out with the WHY-2000 pressure testing machine (as shown in Figure 6, from China University of Geosciences (Beijing)), and the loading rate was 20 mm/min. The sensor layout of the text block is shown in Figure 7.

## 3. Analysis of Test Results

### 3.1. Analysis of Sound Time of Test Block

**Figure 8:**
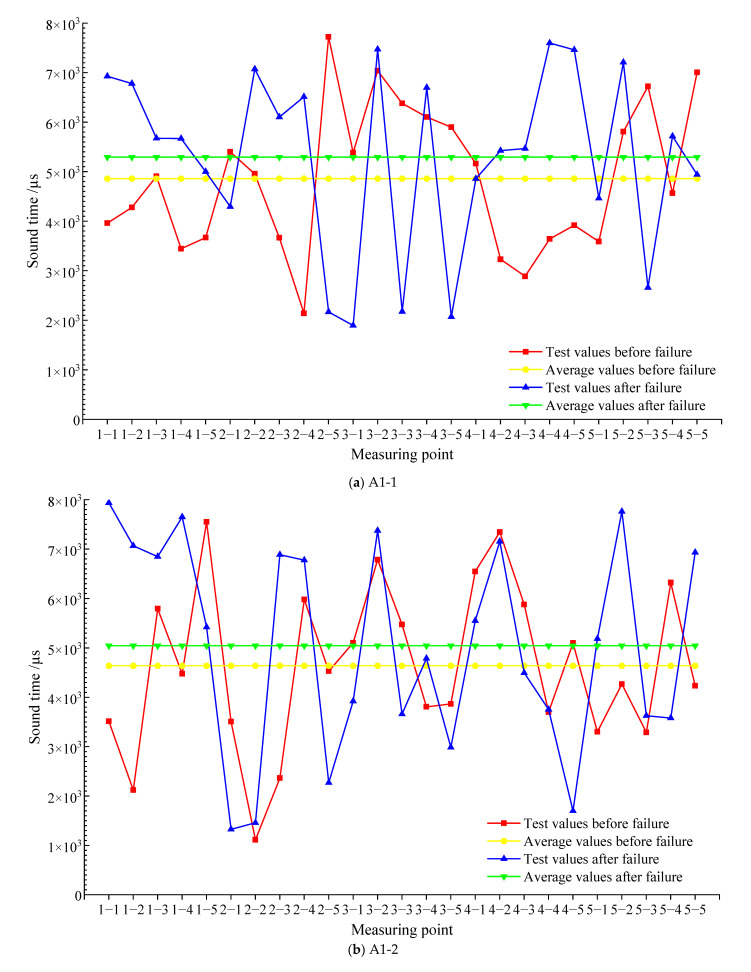

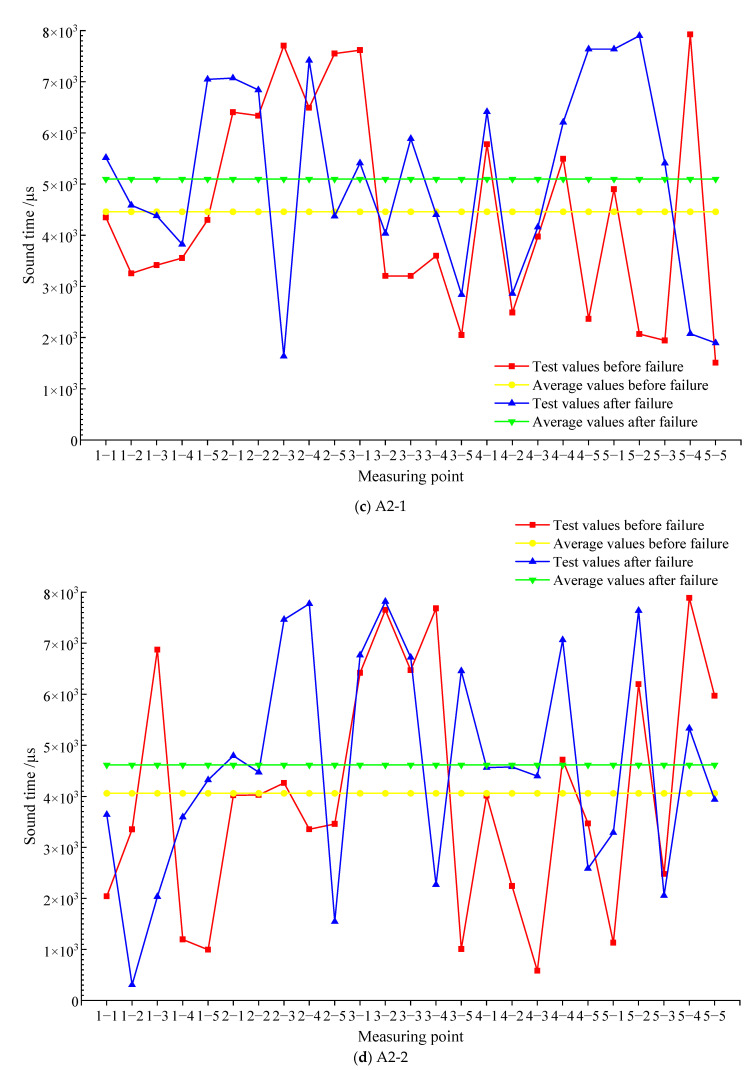
The sound time value before and after the failure of each test block.

Figure 8 shows the sound time value of each measuring point before and after the failure of each test block and the average value of the sound time before and after the failure. As shown in Figure 8a, the average sound time value of test block A1-1 at each measuring point before destruction is 4859.1 μs, the average value of sound time at each measuring point after destruction is 5293.1 μs, the pre-damage average was 91.9% of the post-damage average, and the root-mean-square deviation of test block A1-1 before and after the damage is 2748.4 μs. Among the 25 measuring points, the sound time value of 17 measuring points before destruction is less than the sound time value after destruction, and the sound time value of most measuring points is greater than the sound time value before destruction. As a result, the average value of sound time after the destruction of test block A1-1 is obviously greater than the average value of sound time before destruction. As shown in Figure 8b, the average value of sound time of test block A1-2 at each measuring point before destruction is 4639.3 μs, and the average value of sound time at each measuring point after destruction is 5045.7 μs, the pre-damage average was 91.9% of the post-damage average, and the root-mean-square deviation of test block A1-2 before and after the damage is 2354.7 μs. The sound time value before the destruction of 14 measuring points is less than the sound time value after the destruction, and the sound time value of most measuring points after the destruction is greater than the sound time value before the destruction, so the average value of the sound time after the destruction of block A1-2 is greater than the average value of the sound time before the destruction. As shown in Figure 8c, the average sound time value of test block A2-1 at each measuring point before destruction is 4459.9 μs, the average value of sound time at each measuring point after destruction is 5099.3 μs, the pre-damage average was 87.5% of the post-damage average, and the root-mean-square deviation of test block A2-1 before and after the damage is 2689.5 μs. There are 21 detection points whose sound time value before destruction is less than that after destruction, and the sound time value of most detection points is greater than that before destruction, so the average value of sound time after the destruction of test block A2-1 is greater than that before destruction.

As shown in Figure 8d, the average sound time value of test block A2-2 at each measuring point before destruction is 4061.4 μs, the average value of sound time at each measuring point after destruction is 4618.2 μs, the pre-damage average was 87.9% of the post-damage average, and the root-mean-square deviation of test block A2-1 before and after the damage is 2698.1μs. In the test block, the sound time value after the destruction of 17 measuring points is greater than the sound time value before the destruction, so the average value of the sound time after the destruction is greater than the average value of the sound time before the destruction.

Above all, whether for the SCC test block after curing for 7 days or the SCC test block after curing for 28 days, the average value of sound time of the measured points before the destruction is less than the average value after the destruction, but the difference between the average value of sound time of the measured points before and after the destruction of each test block is small; this indicates that although internal defects occur in the test block after destruction, they are not sensitive to the influence of the average value of sound time. Combining Figure 8 with root-mean-square deviation analysis, the average value of sound time of some measuring points is significantly larger than that before the destruction. In theory, after the failure of the test block under the uniaxial compression test, defects and cracks appear in some structures. When ultrasonic encountered defects and cracks, scattering and reflection occurred. Ultrasonic would bypass defects and cracks and change the original propagation path.

### 3.2. Analysis of Amplitude Value of Test Block

**Figure 9:**
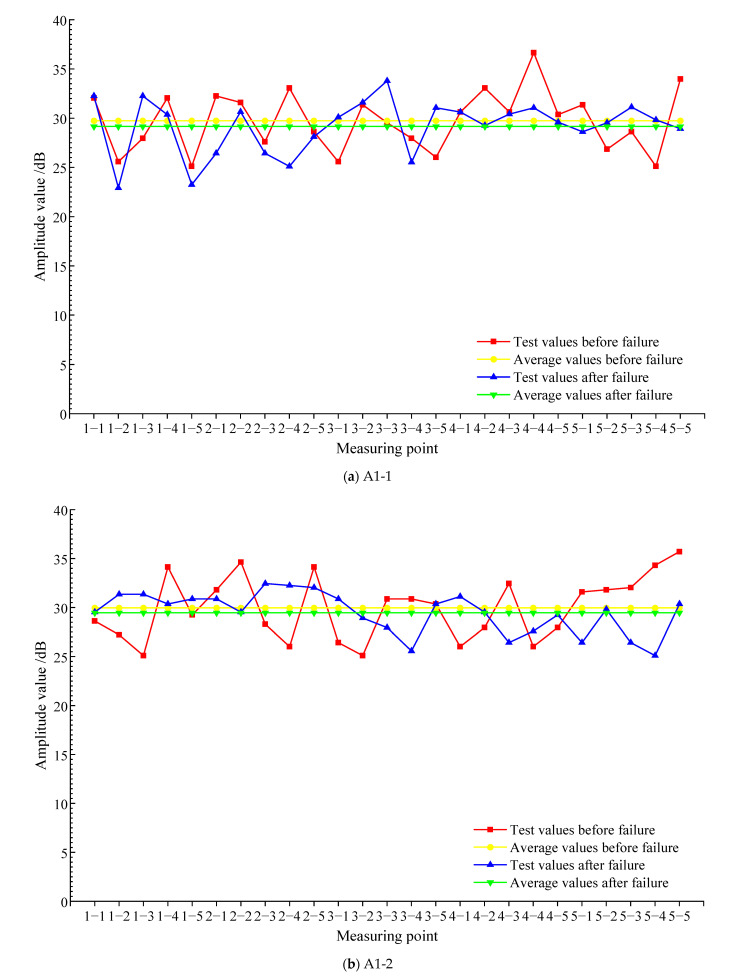

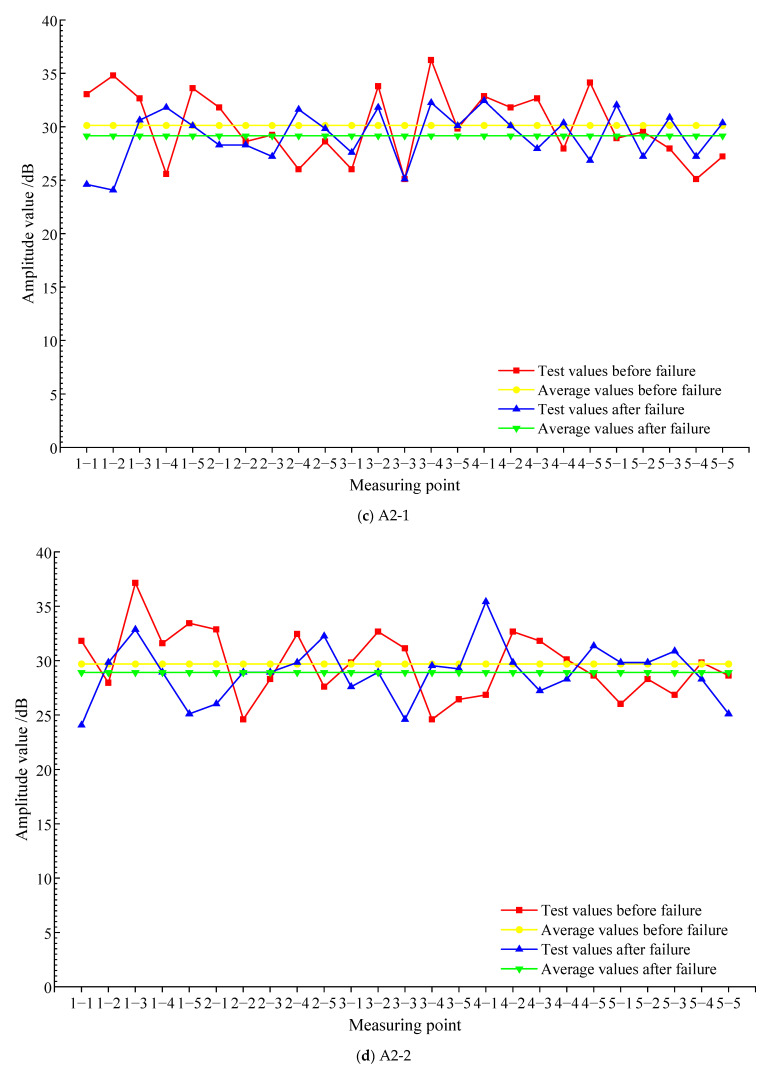
The amplitude value before and after the failure of each test block.

Figure 9 is the average value of the amplitude of each measuring point before and after the failure of each test block. According to Figure 9a, the average value of the amplitude of each measuring point before the failure of test block A1-1 is 29.74 dB, the average value of the amplitude of each measuring point after the failure is 29.16 dB, and the root-mean-square deviation of test block A1-1 before and after the damage is 3.51 dB. In each measuring point, the amplitude of 15 measuring points before failure is greater than that after failure, and the amplitude of most measuring points after failure is less than that before failure, resulting in the average value of the amplitude of A1-1 after failure being less than the average value of the amplitude before failure. According to Figure 9b, the average value of the amplitude of A1-2 before failure is 29.96 dB, the average value of the amplitude of each measuring point after failure is 29.46 dB, and the root-mean-square deviation of test block A1-2 before and after the damage is 4.34 dB. The amplitude of nearly half of the measuring points is smaller than that before failure, and the average value of A1-2 after failure is smaller than that before failure. It can be seen from Figure 9c that the average amplitude of each measuring point of test block A2-1 before failure is 30.13 dB, the average amplitude of each measuring point after failure is 29.16 dB, and the root-mean-square deviation of test block A2-1 before and after the damage is 4.06 dB. The amplitude of 14 measuring points before failure is greater than that after failure, and the amplitude of the remaining measuring points before failure is less than that after failure. The average amplitude of A2-1 after failure is smaller than the average value of amplitude before failure. From Figure 9d, it can be seen that the average amplitude of each measuring point of test block A2-2 before failure is 29.69 dB, the average amplitude of each measuring point after failure is 28.91 dB, and the root-mean-square deviation of test block A2-2 before and after the damage is 4.44 dB. The amplitude of most measuring points after the failure of the text block is smaller than that before failure so the average amplitude after failure is smaller than that before failure. Above all, whether for the SCC test block after curing for 7 days or the SCC test block after curing for 28 days, the average value of the amplitude of the measured points after the failure is smaller than the average value before the failure, but the difference between the average value of the amplitude of the measured points before and after the failure of each test block is smaller. Combining Figure 9 with root-mean-square deviation analysis, the amplitude of some measured points is significantly smaller than that before the failure. This is because the defects and cracks in the structure will lead to scattering and reflection during the ultrasonic wave propagation, and the ultrasonic wave will attenuate obviously, which will lead to the amplitude of some measuring points becoming smaller.

### 3.3. Analysis of Sound Velocity of the Text Block

**Figure 10:**
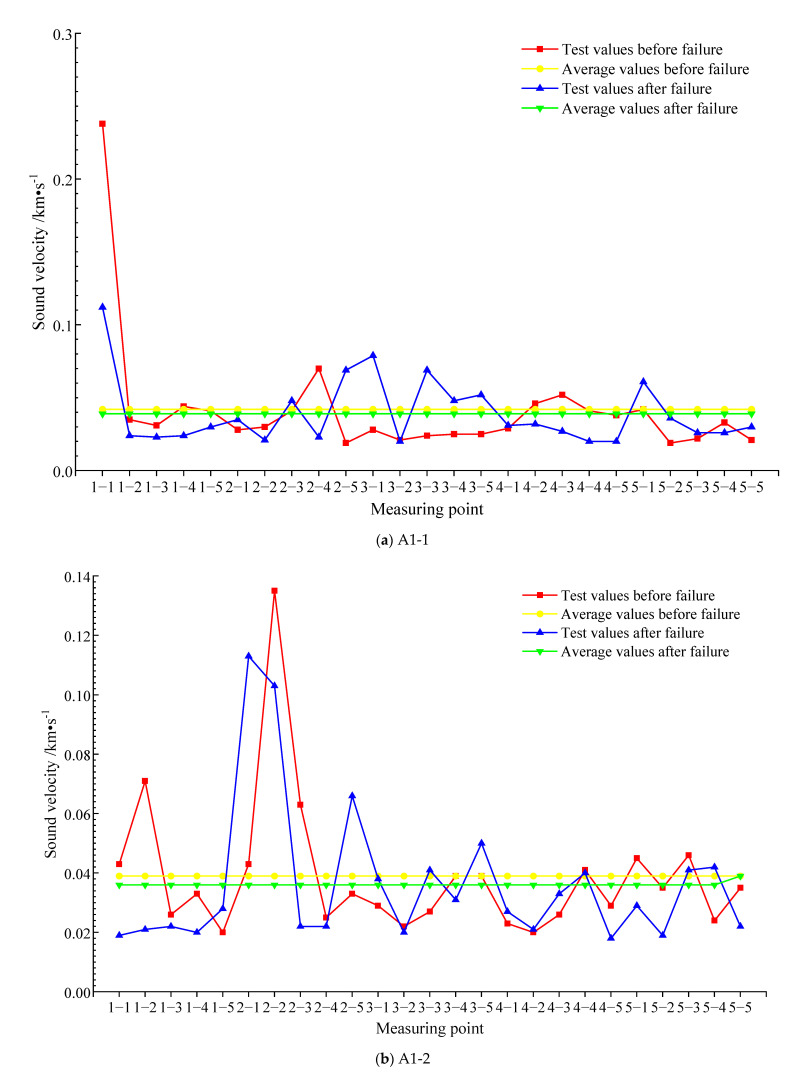

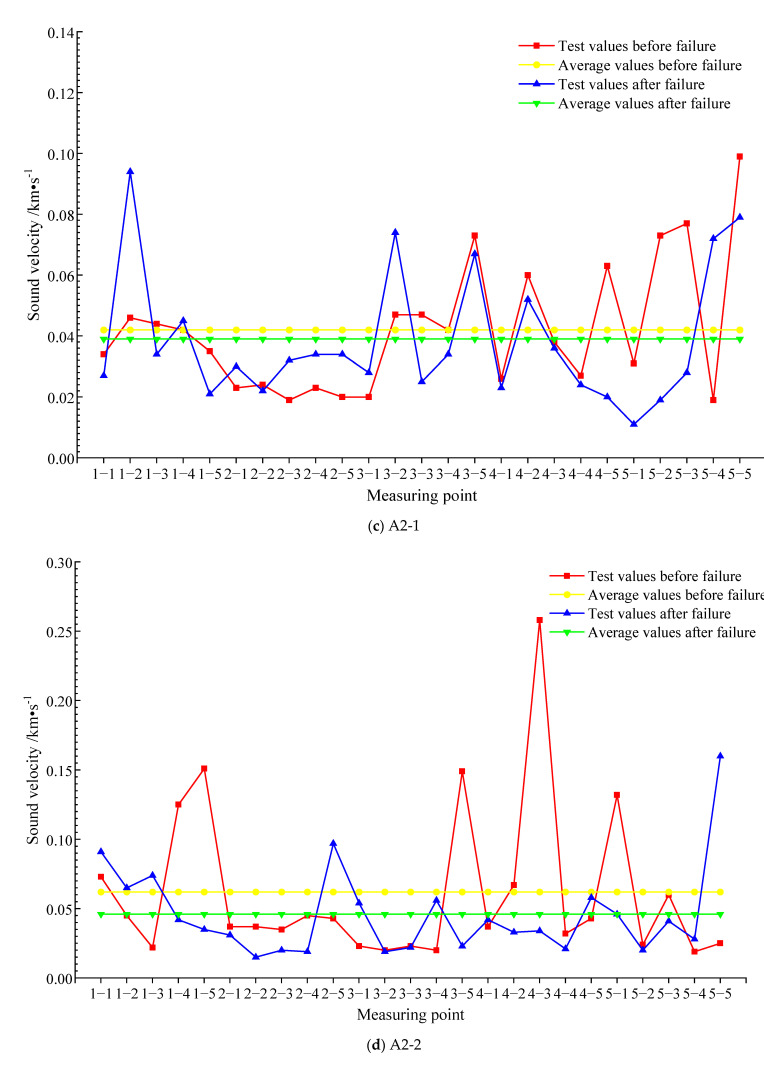
The sound velocity before and after the failure of each test block.

Figure 10 shows the average value of the sound velocity of each measuring point before and after the failure of each test block. It can be seen from Figure 10a that the average sound velocity of each measuring point of test block A1-1 before failure is 0.042 km/s, the average sound velocity of each measuring point after failure is 0.039 km/s, and the root-mean-square deviation of test block A2-1 before and after the damage is 0.034 km/s. In all measuring points, more than half of the sound velocity after failure is less than that before failure, and the average sound velocity of test block A1-1 before failure is greater than that after failure. It can be seen in Figure 10b that the average value of the sound velocity of each measuring point of test block A1-2 before failure is 0.039 km/s, the average value of the sound velocity of each measuring point after failure is 0.036 km/s, and the root-mean-square deviation of test block A2-1 before and after the damage is 0.023 km/s. The average value after failure is 92.3% of the average value before failure. The average value of the sound velocity of 18 measuring points after failure is less than that before failure. The average value of the sound velocity of test block A1-2 before failure is greater than that after failure. It can be seen from Figure 10c that the average sound velocity of each measuring point of test block A2-1 before failure is 0.042 km/s, the average value of the sound velocity of each measuring point after failure is 0.039 km/s, and the root-mean-square deviation of test block A2-1 before and after the damage is 0.025 km/s. The average value of sound velocity before and after the failure of the text block is the same as that of A1-1. The average value of sound velocity before failure of A2-1 is greater than that after failure. According to Figure 10d, the average value of sound velocity after the failure of 16 measuring points of A2-2 is less than that before failure. The average value of the sound velocity of each measuring point before failure is 0.062 km/s, and the root-mean-square deviation of test block A2-1 before and after the damage is 0.069 km/s. The average value of the sound velocity of each measuring point after failure is 0.046 km/s, and the average value after failure is 74.2% of the average value before failure. The average value of the sound velocity of test block A2-2 after failure is smaller than the average value before failure.

Above all, whether for the SCC test block after curing for 7 days or the SCC test block after curing for 28 days, the average value of the sound velocity after the destruction of each measuring point is smaller than the average value before the destruction. Combining Figure 10 with root-mean-square deviation analysis, the sound velocity value of some measuring points is significantly smaller than that before the destruction. This is because the internal medium is more uniform before the failure of the SCC test block. Therefore, the ultrasonic wave propagates at a relatively high speed inside the test block. However, in the later stage, due to the failure of the text block, defects appear in part of the structure of the text block, resulting in the noncompactness of the structure of part of the text block. Therefore, the sound velocity value of some measuring points will decrease significantly.

### 3.4. Analysis of Abnormal Values of Measuring Points

**Figure 11:**
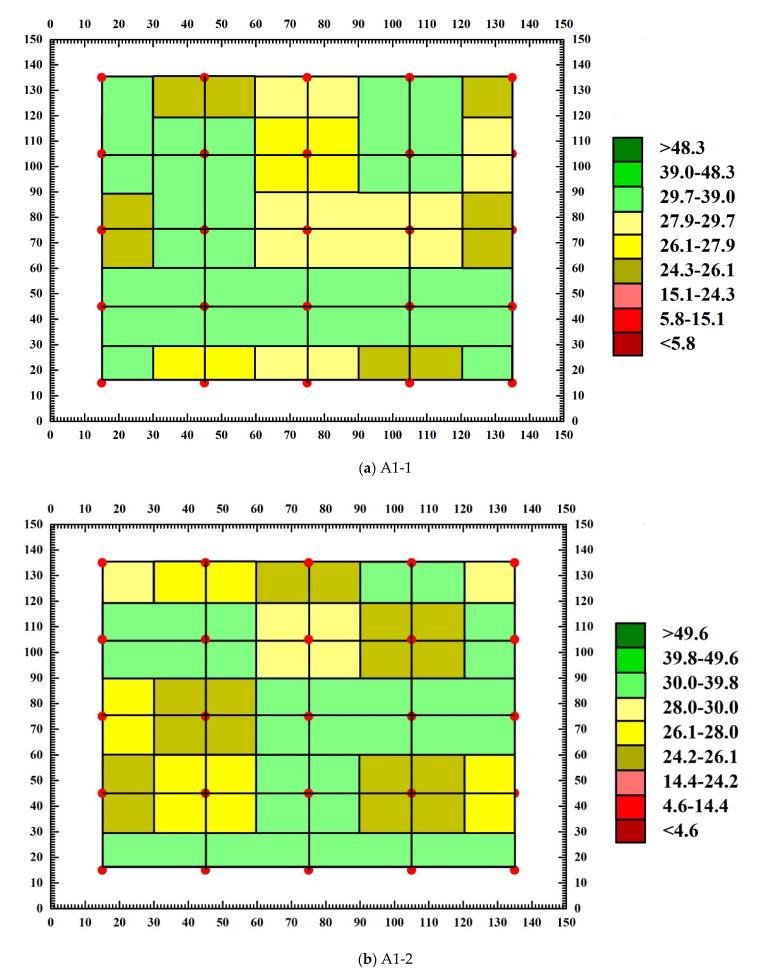

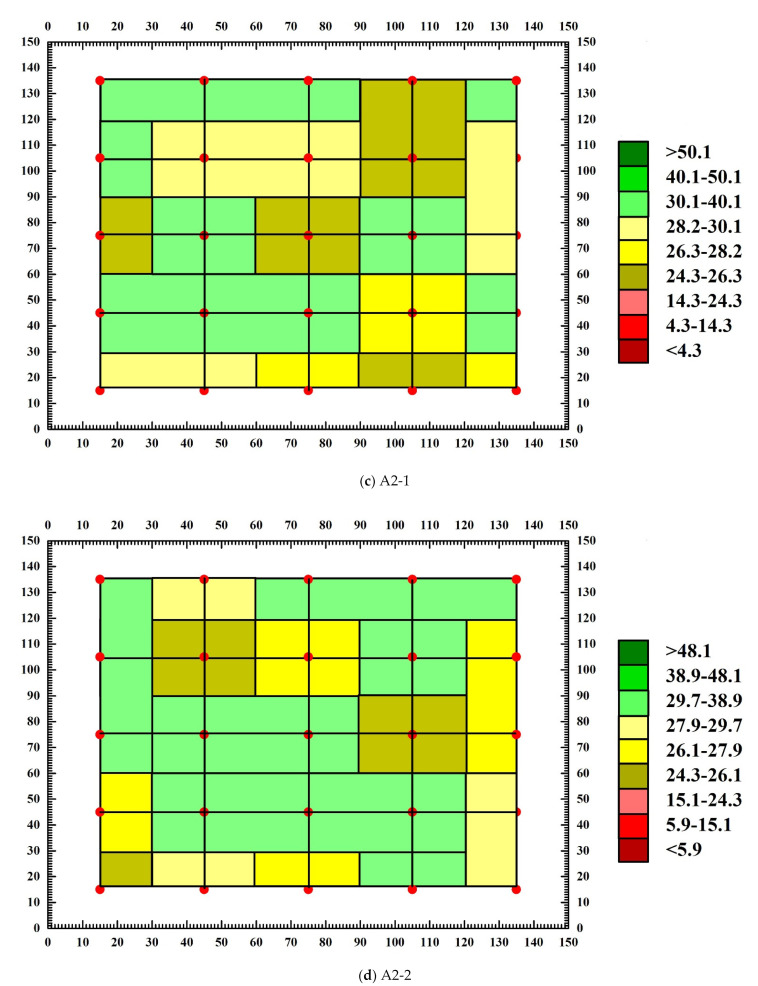
Amplitude chromatogram of test block before destruction.

**Figure 12:**
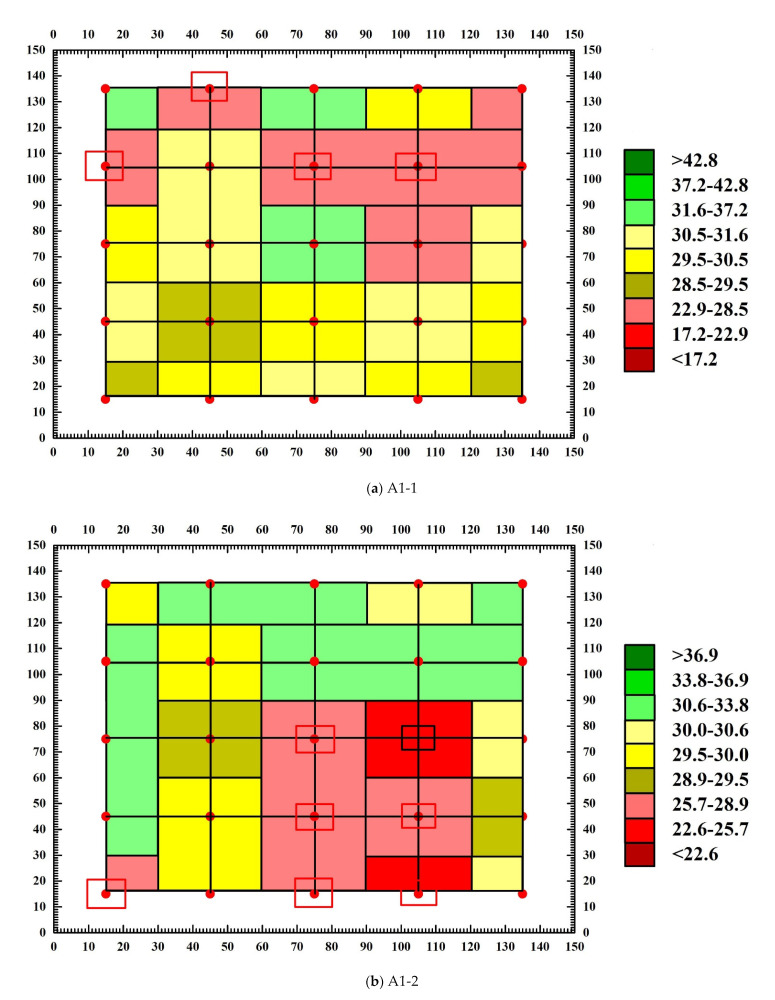

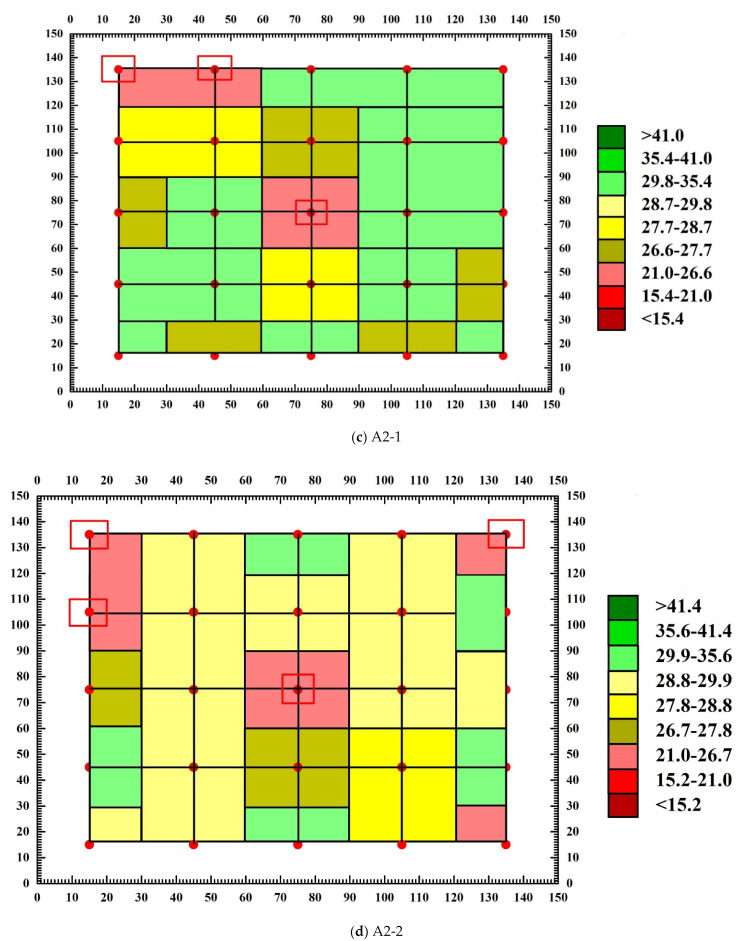
Amplitude chromatogram after test block destruction.

**Figure 13:**
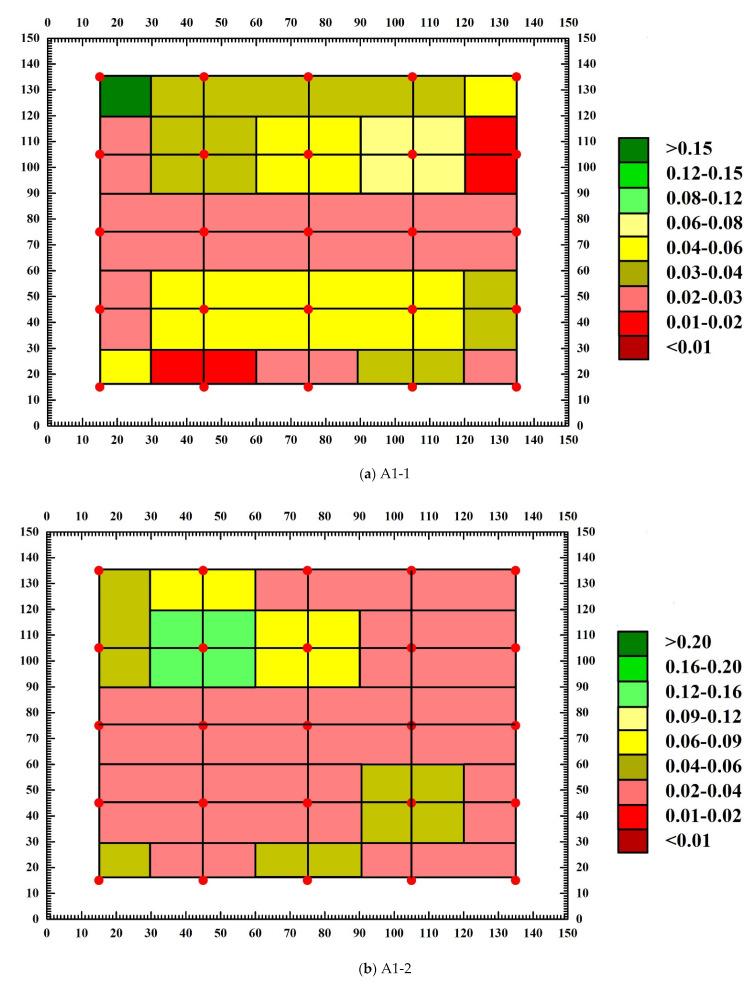

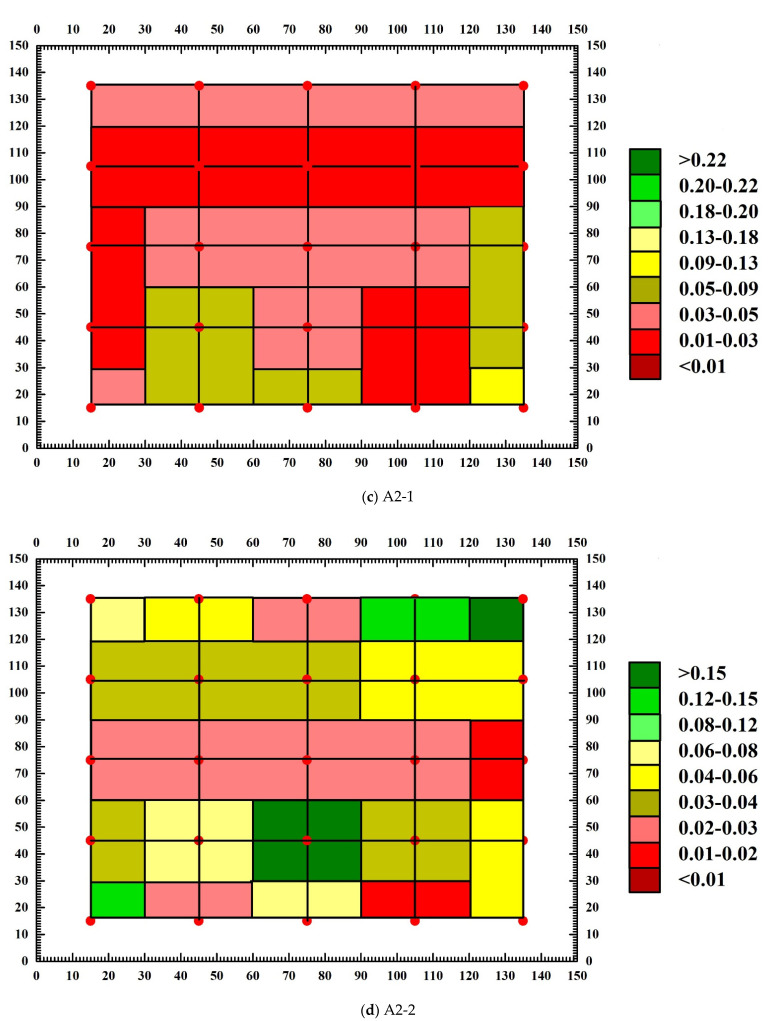
Sound velocity chromatogram before test block destruction.

**Figure 14:**
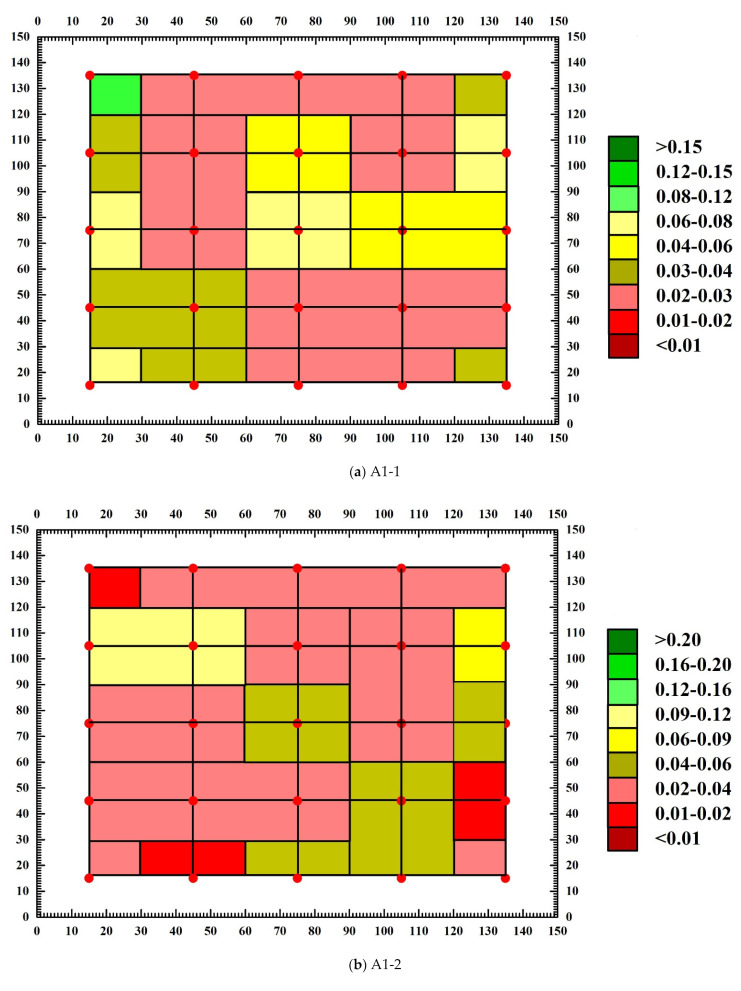

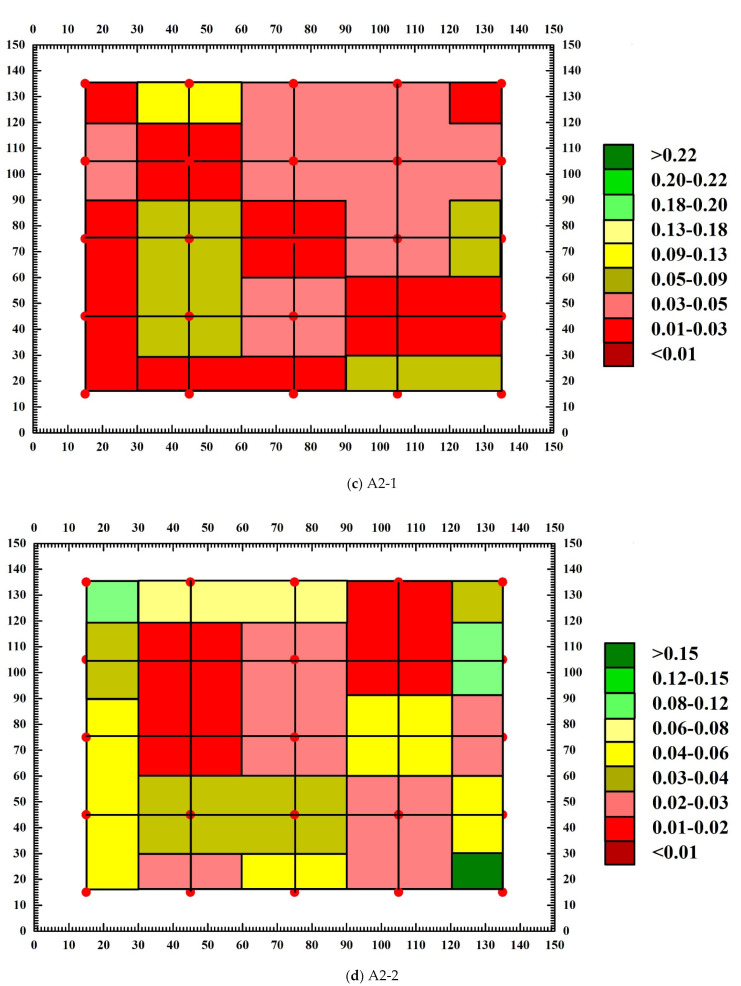
Sound velocity chromatogram after test block destruction.

Through the calculation of concrete sound wave detection and analysis software, the sound velocity chromatogram and amplitude chromatogram before and after the destruction of each SCC test block are obtained, and the abnormal measuring points and abnormal values of the text block are judged. Figure 11 and Figure 12 are the amplitude chromatogram before and after the destruction of the text block, and Figure 13 and Figure 14 are the sound velocity chromatogram before and after the destruction of the text block. It can be seen from Figure 11 and Figure 12 that before the uniaxial compression test, the amplitude of each measuring point of each test block has no abnormal value, but after the destruction of test block A1-1, it is calculated that the abnormal judgment value of a single point is Vo1 = 0.363 km/s, Ao1 = 28.45 dB, the adjacent abnormal judgment value is Vo2 = 0.177 km/s, Ao2 = 29.59 dB, and the amplitude of measuring points 1-2, 2-1, 2-3, and 2-4 are abnormal (the abnormal measuring points have been marked on the diagram); the abnormal values are 22.92 dB, 26.44 dB, 26.44 dB, and 25.11 dB. After the test block A1-2 is damaged, the single point abnormal judgment value is Vo1 = 0.007 km/s, Ao1 = 28.88 dB, the adjacent abnormal judgment value is Vo2 = 0.010 km/s, Ao2 = 29.51 dB, and the amplitude of test points 3-3, 3-4, 4-3, 4-4, 5-1, 5-3, and 5-4 are abnormal; the abnormal values are 27.96 dB, 25.58 dB, 26.44 dB, 27.60 dB, 26.44 dB, 26.44 dB, and 25.11 dB. After test block A2-1 is damaged, the single point abnormal judgment value Vo1 = 0.005 km/s, Ao1 = 26.61 dB, adjacent abnormal judgment value Vo2 = 0.014 km/s, Ao2 = 27.78 dB, and the amplitude of test points 1-1, 1-2, and 3-3 are abnormal; the abnormal values are 24.61 dB, 24.08 dB, and 25.11 dB. After the A2-2 test block is damaged, the judgment value of single point abnormality is Vo1 = 0.738 km/s, Ao1 = 26.74 dB, the judgment value of adjacent abnormality is Vo2 = 0.396 km/s, Ao2 = 27.89 dB, and the amplitude of measurement points 1-1, 1-5, 2-1, and 3-3 are abnormal; the abnormal values are 24.08 dB, 25.11 dB, 26.02 dB, and 24.61 dB. It can be seen from Figure 12 and Figure 13 that there is no obvious abnormality in the sound velocity value before and after the failure of the text block. Through the analysis of the above data, it can be seen that after the failure of each compact concrete test block under the uniaxial compression test, defects and cracks will appear in some structures, resulting in the uncompact structure in some areas. When ultrasonic waves encounter defects and cracks, they will be scattered and reflected, and attenuated significantly. The ultrasonic waves will bypass the defects and cracks and change the original propagation path. Therefore, after the failure of the test block, the defects of the internal structure will cause the amplitude of some measuring points to be abnormal, while the sound velocity value is not abnormal.

**Figure 15:**
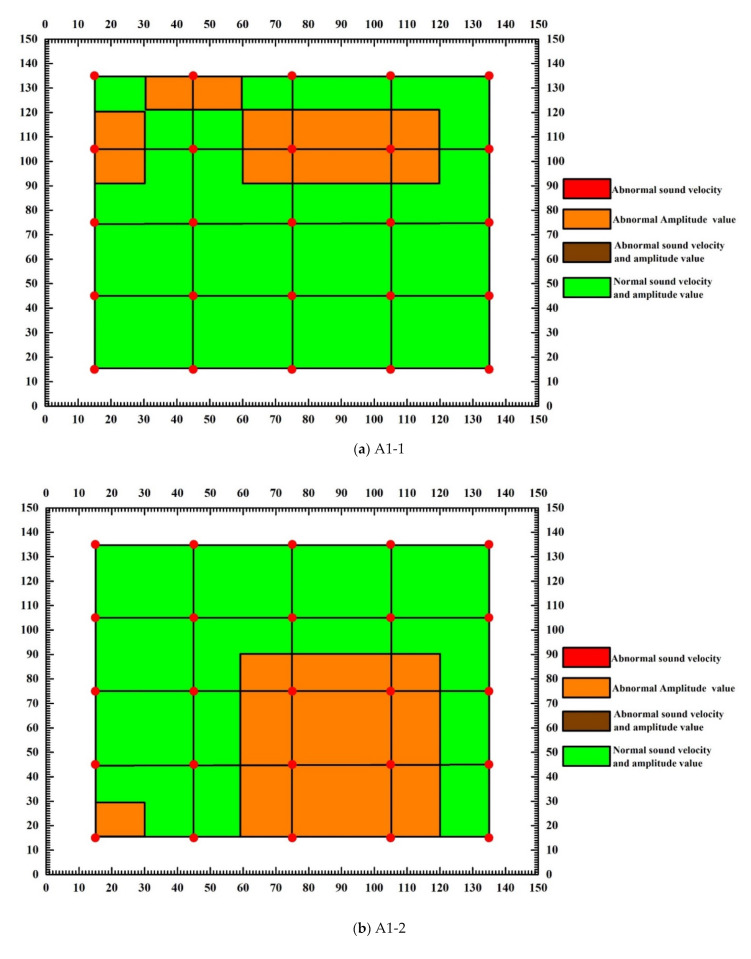

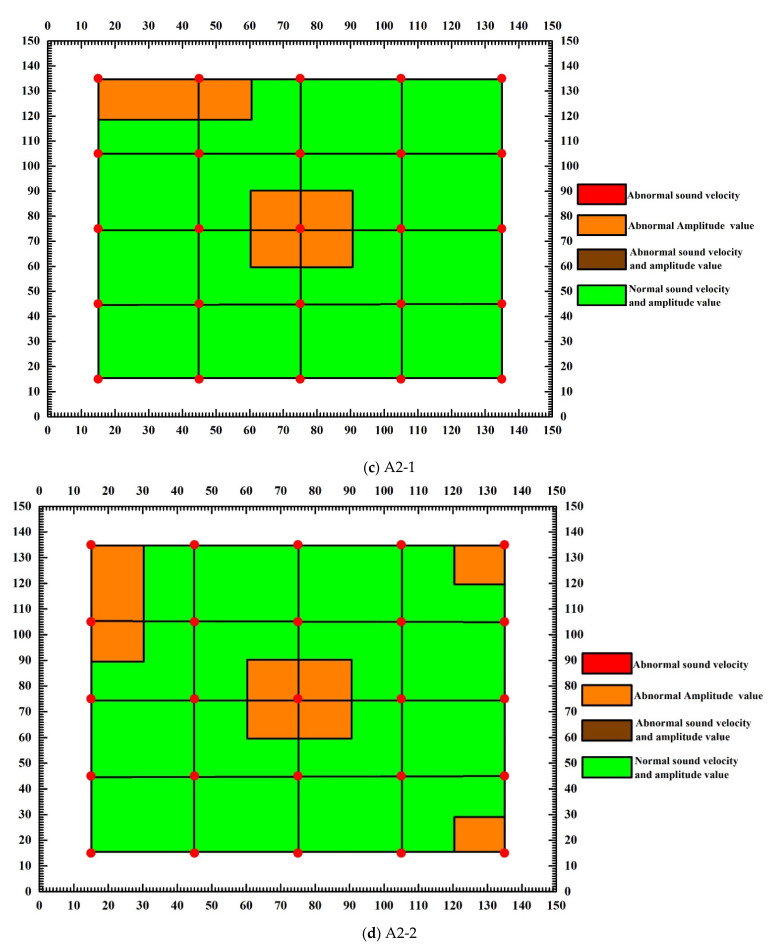
Defect distribution after the failure of each test block.

Figure 15 is the test block defect distribution diagram obtained after the calculation and analysis of the above abnormal measurement points. Before the failure of the text block, the sound velocity amplitude is normal, while the acoustic parameter value is abnormal after the failure. The area with a color anomaly in the figure is the part with the abnormal amplitude value and sound velocity value of the text block. The figure shows that the abnormal value of the block is mainly amplitude value, there are no obvious abnormal sound velocity values, the area of the abnormal amplitude of block A1-1 is concentrated between rows 1-2 and the area of the abnormal amplitude of block A1-2 is concentrated between rows 3–5, the area of the abnormal amplitude of block A2-1 is only concentrated in the top and center of the block, and of the block, A2-2 is concentrated in the center and edge of the block.

## 4. Conclusions

Through the ultrasonic nondestructive testing method, we studied the failure process of SCC under the uniaxial compression test, and the following conclusions were obtained:

i. An ultrasonic testing analyzer was used to study the variation of ultrasonic parameters of SCC before and after uniaxial compression test failure; the evolution law of internal defects of SCC after failure was obtained, which provided a theoretical basis for the application of ultrasonic testing technology in the field of SCC.
ii. After curing the SCC test block for 7 days and 28 days, the sound value before and after the failure had the following rules: The average value of sound time before and after the failure of each measuring point is smaller than that after the failure, but the difference between the average value before and after the failure is small. Defects and cracks appeared in some structures, the ultrasonic propagation path was longer than before the failure, and the sound time value of some measuring points was significantly larger than before the failure.
iii. The amplitude before and after the failure of the test block has the following rules: The average value of the measured points after the failure is smaller than the average value before the failure. Structural defects and cracks cause scattering and reflection during the ultrasonic wave propagation, the ultrasonic wave shows obvious attenuation, and the amplitude of some measured points is significantly smaller than that before the failure.
iv. The sound velocity values before and after the failure of the test block have the following rules: The average value of the sound velocity after the failure of each measuring point is smaller than the average value before the failure. The test block is damaged, and some of the structures are defective, resulting in the uncompacted structure of part of the block, and the sound velocity value of some measuring points is significantly smaller than before the destruction.
v. During the SCC ultrasonic testing process, the ultrasonic velocity was affected by many factors. In the subsequent testing process, the influence of these factors must be reduced.

## Figures and Tables

**Table 1:** Performance index of each raw material.

	Performance	Bulk Density	Performance Density	Fineness Modulus	Initial Setting Time	Final Setting Time	Fineness
Raw Material							
Ordinary Portland cement		----	3100 kg/m^3^	----	>150 min	<240 min	----
River sand		1600 kg/m^3^	2600 kg/m^3^	2.3–3.0	----	----	----
Fly ash (grade 1)		----	2400 kg/m^3^	----	----	----	----
Gravel		1500 kg/m^3^	1600 kg/m^3^	----	----	----	----
Polycarboxylic water reducer		----	----	----	----	----	----

**Table 2:** Mix proportion of SCC.

Raw Material	Cement	Fly Ash	Sand	Gravel	Water	Water Reducer
Mix proportion	20.18 kg	5.69 kg	40.74 kg	25.87 kg	9.31 kg	0.164 kg

**Table 3:** Measured and specified values of performance of SCC.

Index	Test Performance	Numerical Range	Measured Value
Slump flow (mm)	Filling property	SF2 (660–755)	720
Extension time T500 (s)	Filling property	2–5	4.2
V-funnel time (s)	Filling property	9–20	18.4
H2/H1	Interstitial permeability	≥0.8	1.0

## Data Availability

The data presented in this study are available in Supplementary Material figure data.

## Supplementary Materials

The following supporting information can be downloaded at: https://www.mdpi.com/article/10.3390/ma15134412/s1. The information for Figures S1–S3 can be found in an excel file named figure data in the Supplementary Materials.
